# Public primary healthcare service network planning in Finland: a document analysis of principles, justifications, and future directions

**DOI:** 10.1080/02813432.2026.2718263

**Published:** 2026-08-20

**Authors:** Visa Väisänen, Liina-Kaisa Tynkkynen, Timo Sinervo

**Affiliations:** ^a^ Finnish Institute for Health and Welfare, Welfare State Research Unit, Helsinki, Finland; ^b^ Faculty of Social Sciences and Business Studies, Department of Health and Social Management, University of Eastern Finland, Kuopio, Finland

**Keywords:** Primary healthcare, service network, planning, health system reform, document analysis

## Abstract

**Background:**

Maintaining accessible primary healthcare (PHC) services requires evidence-informed decision-making, especially during health system reforms, which can implicitly or explicitly affect PHC service networks. Following a large-scale administrative reform, Finnish wellbeing services counties are reshaping their local service networks. We investigated the principles guiding PHC service network planning, justifications for service network adjustments, and views on future PHC service provision.

**Methods:**

Publicly available policy documents (*n* = 31) concerning PHC service networks were systematically identified and extracted from county websites. We followed the READ approach and utilized document analysis with inductive content analysis.

**Results:**

Public PHC service network planning focused on travel times and accommodation for external, mainly population-related factors. All counties were also implementing tiered service provision. Service network changes were justified by workforce challenges, particularly in remote areas, and poor condition of premises. Additionally, centralization was argued to enable higher standards of care. Financial aspects were not strongly emphasized. Future PHC centered around digital and mobile care services, which were seen as prerequisites for reducing physical services, while detailed long-term planning was largely absent.

**Conclusions:**

The counties’ service network analyses highlighted the complexity of planning and a concurrent balancing act between accessibility, service quality, workforce availability, and physical premises. The strenghtening of acccessibility and availability was emphasized, while the service provision was adapted to the available resources. Our findings can support evidence-informed planning and implementation of health system reforms which affect PHC and provide a basis for further research on PHC service networks and accessibility.

## 1. Introduction

Accessible and timely primary healthcare (PHC) services are central in responding to the numerous challenges facing health systems all over the world [1]. These systemic pressures include ageing populations [2], continuing urbanization [3], workforce shortages [4], and the increasing burden of chronic disease [5] as well as mental health disorders [6]. Both national and local governments have a responsibility for ensuring the accessibility of PHC, including the dimensions of availability and accommodation (i.e. care supply, waiting times, and travel distances) [7,8]. Consequently, efficient decision-making and service planning are crucial for improving and maintaining PHC access for all populations, including people in marginalized positions [9,10].

One central aspect of ensuring accessible PHC services is decision-making and planning related to service networks, with local actors deciding how to ensure comprehensiveness. Countries that rely on private service provision can utilize various financial and non-financial incentives or regulations to guide the placement of providers [11,12]. Meanwhile, health systems with at least some level of public PHC provision offer opportunities for more direct involvement. For instance, in Denmark, regions’ own or contracted GP clinics can supplement service provision in identified underserved areas [13], and in Finland, the wellbeing services counties (WSCs) can decide themselves where PHC services are located.

PHC service network planning is scarcely researched, with little information on the different guidelines and principles public actors have formulated and how they utilize and prioritize these measures. Windle and colleagues have examined the use of evidence in regional PHC planning [14,15], but to our knowledge, no research on PHC service network planning of public actors exists. In operations research, various approaches to health service network optimization have been developed [16,17]. However, it is unknown to which extent policymakers and service network planners utilize these algorithmic models (if at all) and whether their use is transparently documented. Additionally, policymakers must contend with political realities, parallel private service provision, national regulations, ownership and physical condition of premises, and health workforce availability. Consequently, a significant research gap in PHC service network planning conducted by public actors was identified.

Finland has a universal health care system based on health and social care services organized by 21 WSCs (and the capital city of Helsinki), which receive general funding from the government in accordance with a needs-based allocation formula [18]. The WSCs can freely allocate this funding to different sectors. The WSCs are self-governing, led by county councils consisting of democratically elected officials. Public PHC services are offered in health care centers with varying service portfolios, where the initial point of contact is a registered nurse acting as a gatekeeper to salaried physicians [18]. Larger health centers utilize multidisciplinary team models, integrating both health and social care services [19]. While most residents do not yet have a personal GP, there is an accelerating trend toward personal doctor-nurse working pairs [20]. Due to the occupational healthcare (OHC) system, which provides better access to ambulatory care services for majority of the employed, public PHC services cater mostly to older people, unemployed people, and children [21]. Higher education students have their own services organized by Kela [18]. Notably, outside of the non-urgent care guarantee legislation (3 months to a GP after care needs assessment), no national service promise on accessibility exists, and instead, the Finnish constitution mandates that public authorities guarantee ‘adequate’ services for everyone [22].

Health system reforms often have explicit or implicit aims, which can include local structural reforms, as well as strengthening or increasing the sustainability of PHC services [23]. In Finland, a large-scale administrative reform was implemented in 2023, which transformed the structures of political decision-making and steering [18,24]. Notably, the reform scope excluded parallel PHC systems (e.g. OHC), maintaining existing inequalities in care access [21,25]. The reform originated from a need for ‘wider shoulders’: ensuring that service organizers have sufficient financing and integrated service provision capacity to efficiently meet the growing care demands caused by population ageing. The reform aimed to improve access to care, ensure the quality of services, control the increasing health and social care related costs, and shift the focus to primary services [26].

The reform implementation has taken place in challenging circumstances, including post-pandemic care backlogs, high inflation and the subsequent wage increases, staff salary harmonization, national financial struggles, and potential historic underreporting of municipal costs [18]. Consequently, the WSCs have commenced their operations in significant budgetary deficits [18,27], contributing to rapid local structural reforms and cost-containment measures that are also impacting PHC service networks [28]. The difficult conditions and the urgency to implement local changes might affect planning and decision-making related to PHC service networks. Considering the official reform aim of strengthening PHC services (often referring to increasing their emphasis and shifting the focus from specialized services), it is imperative to analyze the strategic planning of these new actors.

The Finnish reform has so far resulted in significant PHC service network changes, induced by the accumulated service reform needs and austerity caused by significant budgetary deficits. Recent analysis indicates that the health center unit closures (108 in 82 municipalities) have focused on areas with denser existing PHC networks, largely avoiding underserved areas [28]. However, these changes have generated concern among both the general population [29] and the health workforce [30], likely due to the perceived erosion of historically and culturally rooted identities associated with local municipal-based services, such as the concept of a healthcare center [28,30].

PHC is systemic in nature and shaped by different functions of the overall health system [31,32]. The Health System Performance Assessment (HSPA) framework by WHO and European Observatory on Health System and Policies, which we utilize as a conceptual reference in this study, outlines these various functions [31]. It includes governance by both local and national actors, financing, resource generation, and service delivery. Consequently, when planning or evaluating reforms affecting health systems, and often directly or indirectly also PHC services, these different effect pathways should be considered. For example, the placement of health workers, supply and demand of care, and physical infrastructure can impact service delivery. Last, effective leadership and governance, at all levels of administration, are crucial for promoting evidence-informed decision-making and planning in public PHC, including strategic policy guidance and optimal allocation of limited resources [31]. High-quality governance is also key during significant system-level transformations [33].

In addition to illuminating the local planning and decision-making processes following a large-scale reform, relevance for international audiences emerges particularly in countries planning similar reforms. Finland acts as a ‘forerunner’ in many ways with its sparsely living and ageing population, but also in terms of modern PHC service models, including multidisciplinary teams and advanced digitalization. As such, the views of the Finnish WSCs on the development of PHC networks and services can provide insights for countries attempting to strengthen or safeguard their PHC services.

The overarching aim of this study was to investigate the PHC service network planning of new regional actors following a large-scale health system reform. This research can help understand the measures local planners and policymakers utilize for improving or maintaining the performance of the PHC services, under the given budget constraints. The principles and justifications of WSCs can also illuminate the prioritization of different factors, such as the balance between geographical accessibility and service availability, while accounting for various external limitations to service provision. Moreover, local planning processes and decision-making are central for population health outcomes, as they directly shape the accessibility of PHC services as well as potential barriers to care utilization. Last, our findings provide insights into future research on the design or planning of service networks, also beyond the PHC context. In line with the identified research gaps, three research questions were formulated:

1. What kind of principles guide the planning of WSCs’ PHC service networks?
2. How are the health center unit closures, mergers, and service network adjustments justified in the WSCs’ documents?
3. How do the WSCs view the future of public PHC service networks and service provision?

## 2. Materials and methods

### 2.1. Study design

The Finnish WSCs are mandated by law to produce various strategy documents, and many have additionally published various reports on their service networks. These publicly available policy documents involve close collaboration between elected officials and public servants and can reveal whether decision-making is evidence-informed. In this study, we examine policy documents of WSCs to investigate how they approach service network planning. Document analysis utilizing qualitative inductive content analysis was employed [34,35]. We utilized the READ approach by Dalglish and colleagues [36], which is a systematic framework that provides practical guidance for conducting document analysis in health policy research.

### 2.2. Data collection

Policy documents regarding PHC service provision and service networks were systematically identified and extracted from the websites of WSCs. Relevant policy documents were various service network plans or reports and service provision or premises program plans. If no specific service network documents were found, relevant meeting notes and other strategy documents were extracted. Additionally, to answer the third research question, general strategies or other documents that explicitly indicated a longer time span (e.g. year 2030 or beyond) were also included. While some of these documents (strategies) were mandated by law, the function of the service network analyses appeared to focus on offering baseline suggestions of network reconfiguration for political decision-making.

Inclusion criteria for the documents were: 1) publicly available in the WSC websites or public repositories, 2) included information about PHC service network planning (i.e. principles, changes, decisions, or future outlooks), and 3) concerned the operations of the WSC, with publication dates starting from 1^st^ of January 2023 to the cut-off date for data collection, 22^nd^ of September 2025.

The data sources included in the study (*n* = 31) are presented in Table 1. The sources used varied in length (10 to 334 pages) and form (conventional report, slideshow presentation, or meeting notes). Service network plans, proposals, or reports were often the outputs of planning processes, while the service strategies, meeting notes, and programs were more adjacent to political decision-making, guiding the planning.

**Table 1.** Data sources used and basic demographic information per wellbeing services county (WSC), ordered by descending population size.

Wellbeing services county	Data source(s)	Time frame	Population	Land area (km^2^)
City of Helsinki	1. Service strategy 2. Principles of service network planning - meeting notes	2023–2025 2023–	689,758	215
Pirkanmaa	1. Strategy of the WSC 2. Plan of service network	2023–2025 n.d	546,075	13,249
Southwest Finland	1. Service network plan	2024–2026	483,561	10,666
West Uusimaa	1. Guidelines for developing the service network in accordance with the service strategy	n.d	480,675	4,252
North Ostrobothnia	1. Organization plan 2. Definition and number of necessary health care centers	2023–2025 2023–2025	415,867	36,828
Vantaa and Kerava	1. Service network plan	2024–2034	277,568	269
Central Finland	1. Service network proposal	n.d	274,112	16,042
North Savo	1. Service strategy	2023–2025^a^	247,984	17,344
Satakunta	1. Service network report 2. Service network plan	n.d –2030	213,685	7,821
Päijät-Häme	1. Service strategy 2. Premises program: Analysis of the status and the premises network	2023–2025 2023–2036	204,839	5,713
Central Uusimaa	1. Network plan of services	2024–2030	202,597	1,669
South Ostrobothnia	1. Service network reform	2023–2026^a^	191,513	13,798
Lapland	1. Service organization program and service strategy 2. Service network reform plan	2024–2026 n.d	176,083	92,674
Ostrobothnia	1. Strategy 2. Future and adjusting program	2023–2026 2024	175,829	7,401
Kanta-Häme	1. Service network report	n.d	170,028	5,199
North Karelia	1. Service strategy	2024–2038	162,907	18,791
Kymenlaakso	1. Strategy of the WSC 2. Service structure and service network of the WSC (meeting agenda)	2023–2025 n.d	160,757	4,558
South Savo	1. Service strategy and service provision plan	n.d	131,310	12,652
South Karelia	1. Service strategy 2. Service network report	2023–2025 n.d	125,661	5,326
East Uusimaa	1. Service network plan	2025–2026	99,204	2,703
Kainuu	1. Organization plan	2023–2025	70,960	20,197
Central Ostrobothnia	1. Soite-program 2030	–2030	67,995	5,020

n.d., Not defined; WSC, Wellbeing services county.

^a^Only part of the document concerns this time frame.

*Note*. The capital city of Helsinki is treated as a wellbeing services county.

### 2.3. Data analysis

We utilized the READ approach by Dalglish and colleagues [36], which comprises four steps: 1) readying of materials, 2) extracting data, 3) analyzing data, and 4) distilling the findings. Our document analysis approach was inductive [35].

After readying the materials, the documents were skimmed to identify sections on PHC relevant to the research questions. These sections were then read carefully and analyzed by the first author through open coding, producing condensed notes capturing the topics found. The notes were compared across documents, and reoccurring topics were organized into preliminary categories. Placement of topics into categories was guided by the research questions, where the notes were first grouped into principles (RQ1) and justifications (RQ2), and were then combined into broader categories. The initial categorizations were subsequently discussed, iterated, and refined together with the coauthors. Importantly, the categories (*n* = 10) were mapped according to the research questions with some overlap, reflecting the interconnected nature of the service network planning process. Especially many of the justifications for network changes (RQ2) could also be treated as external factors related to the network planning, as part of the principles guiding PHC service provision (RQ1) and vice versa. While the research approach was inductive, the HSPA framework was used as a guide to help with the categorizations.

To demonstrate the analysis logic utilized and the grouping of topics into categories, a translated excerpt describing the details of service network planning is presented in Table 2. The categories per research question are summarized in Table 3. Selected quotes from the documents illuminating the categories are presented later in Table 4.

**Table 2.** An example of the analysis logic utilized showing a translated excerpt concerning service network planning of two health centers in a medium sized WSC located in Southern Finland.

Original excerpt	Condensed notes	Grouping into categories
‘The development prospects for the area in connection with the new tramway preparations must be considered when developing health services, so that the population changes and changes in service needs caused by regional development are taken into consideration. […] In the future, it would be necessary to merge [neighborhood A] and [neighborhood B] health centers into one unit, accounting for the size of the staff. A single unit would ensure better accessibility and reduce vulnerability in terms of personnel. […] The future of the current [neighborhood A] health center is uncertain due to plans to demolish the shopping center. The new combined health center should be located near the future tram line to ensure good accessibility.’	Considering changes in public transport infrastructure Accounting for population changes and service needs (regional developments) Merging of service points due to small staff size and to reduce vulnerability Development of local amenities is relevant Larger units and good location help accessibility	RQ1: Accommodating for external factors (1,2,4)
		RQ1: Travel times and distances (1,5)
		RQ2: Workforce availability (3)
		RQ2: Maintaining high standards of care (5)

RQ1: What kind of principles guide the planning of WSCs’ PHC service networks?

RQ2: How are the health center unit closures, mergers, and service network adjustments justified in the WSCs’ documents?

RQ3: How do the WSCs view the future of public PHC service networks and service provision?

**Table 3.** Overview of the results.

Research question	Category	Summary of findings
1. Principles guiding PHC service planning and provision	1.1 Tiered service provision	PHC services are being restructured into tiers, typically consisting of larger health centers, health stations, and local service points. This enables service centralization while maintaining local accessibility by reducing service portfolios and avoiding closures.
	1.2 Travel times and distances	Travel time and distance metrics are central indicators for evaluating the accessibility impacts of local reforms. In general, the reported changes to geographical accessibility (percentage of population reaching services within X minutes) were small.
	1.3 Accommodating for external factors	Service network planning is strongly guided by population demographics, mobility patterns, and local infrastructure developments. Forecasted changes in service needs, especially those related to ageing, urbanization, and linguistic or cultural requirements, influence planning.
2. Justifications for changes (health center unit closures, mergers, network adjustments)	2.1 Workforce availability	Workforce shortages, recruitment difficulties, and upcoming retirements are major drivers of service network reforms. Small and rural service units are viewed as particularly vulnerable due to staffing challenges, limited resilience, and difficulties in supporting multidisciplinary care models.
	2.2 Maintaining high standards of care	Larger and more centralized units are perceived to improve accessibility, workforce availability, efficiency, multidisciplinary collaboration, continuity, and readiness for complex care. Centralization is justified as a means to maintain quality and availability of care.
	2.3 Premises management and infrastructure	The premises, inherited from previous organizers, are seen as aging, unsuitable, and too costly to invest in, thus driving the service network reforms. WSCs aim to optimize facility use and align investments with forecasted service needs (see population ageing, urbanization).
	2.4 The budget	Financial pressures and budget deficits are generally not presented as the primary justification for the changes. Economic considerations are introduced alongside workforce and infrastructure challenges, while emphasizing continued investment in PHC.
3. Future of PHC service networks and service provision	3.1 Digital care	Digital care is viewed as a key enabler of future PHC provision, with expectations of significant growth in remote consultations. Digital care is perceived to improve accessibility, address workforce constraints, and support service centralization.
	3.2 Mobile care services	Mobile services are increasingly used to provide care in remote areas without maintaining permanent facilities. They are expected to improve accessibility, support more specialized services, and offer flexibility in workforce utilization.
	3.3 Lack of future planning	Most service network plans focus on short-term reforms and provide limited long-term strategic vision beyond year 2030. The current reforms are often presented as the initial phase of ongoing service network development, with further changes anticipated.

**Table 4.** Selected quotes from the analyzed materials illuminating the categories (*n* = 10).

Category	Quotes
1.1 Tiered service provision	‘[Primary healthcare] services are provided at health centers, ‘local’ health stations and, if necessary, as mobile services. Health centers have a wider range of services than local health stations. Local health stations and mobile services are supported by health centers in their operations.’ – Large WSC in southern Finland. ‘The baseline is that each municipality has a health and social services center. These centers are profiled and provide services according to the needs of the area’s residents.’ – Medium sized WSC in southern Finland.
1.2 Travel times and distances	‘Health and social services will remain easily accessible throughout the county, even though three centers will be closed. […] [After centralization] an estimated 98% can receive physical healthcare services within a 30-minute drive.’ – Small WSC in southern Finland. ‘In the current situation, the population is reached fairly well, but if the geographical coverage of the service network is reduced, areas that are already challenging [in terms of accessibility] must be considered.’ – Medium sized WSC in southern Finland.
1.3 Accommodating for external factors	‘Principles affecting the planning of the service network and service selection: The operating environment of the county (e.g. daily activities, commuting, geographical distances, transport connections, educational institutes, pharmacies, other public services, and other wellbeing services counties).’ – Large WSC in eastern Finland.
2.1 Workforce availability	‘Due to high retirement rates and shrinking age brackets, there are challenges in the availability of [healthcare] personnel. There are no longer enough personnel to maintain the service network under the current structure.’ – Large WSC in eastern Finland ‘[By centralizing services to centers and stations] the operational reliability of services increases, as a wider work community is less vulnerable to absences and finding substitute staff is easier. […] Support from a larger work community also facilitates recruitment, enables skills development and diverse career paths, and promotes wellbeing at work.’ – Medium sized WSC in eastern Finland.
2.2 Maintaining high standards of care	‘Sufficiently large units can also be justified on the basis of factors related to client and patient safety, expertise, service quality, and operational reliability, which benefit both clients and staff.’ – Medium sized WSC in western Finland. ‘[Support of a larger work community] enables the development of continuity of care, multidisciplinary work, and ensuring of competencies’ - Medium sized WSC in eastern Finland.
2.3 Premises management and infrastructure	‘Approximately 65% of properties used in PHC are in poor or passable condition. […] The current service network is … in poor technical condition – significant short-term renovation needs have been identified in several locations, for which there are no official plans for yet.’ – Small WSC in eastern Finland. ‘The facilities currently in use in the WSC create the operating conditions for the services provided and tie these services to specific locations where they have previously been provided. Now, with the reform, municipal boundaries have been removed, allowing for a completely new approach to service provision.’ – Medium sized WSC in western Finland.
2.4 The budget	‘The reform’s funding model is highly challenging for the county. […] Despite this, an even greater challenge than funding during this and the next strategy period is the availability of personnel to meet the service needs, even to ensure the provision of legally mandated services.’ – Medium sized WSC in southern Finland.
3.1 Digital care	‘The goal for 2030 is that 50% of [outpatient PHC consultations] will be conducted remotely. Based on this target, we have calculated that in 2030 approximately 20% of current physical visits to outpatient healthcare facilities would be replaced by digital services, freeing up a corresponding proportion of premises for consultations from other areas, for instance.’ – Large WSC in western Finland.
3.2 Mobile care services	‘Digital and mobile services are gradually replacing some proportion of physical services. […] Mobile services are being introduced to areas where the service network has been centralized, the population is ageing, and services are not easily accessible by public transport.’ – Medium sized WSC in southern Finland.
3.3 Lack of future planning	‘The second phase of the service network work will be carried out in spring 2026. At that time, decisions will be made for 2026 and refinements to the service network plan till 2030. The refinements will be influenced by the regular evaluation of the service network, financial situation of the county, establishment of operating and service models, and the need for changes in operations.’ – Large WSC in central Finland.

## 3. Results

### 3.1. Principles of PHC service planning and provision

#### 3.1.1. Tiered service provision

Most WSCs are transitioning to a system with clearly defined tiers of physical services, for instance 1) health center, 2) health station, and 3) service point. The most common configuration was a three-tiered system. A tiered service provision can offer the WSCs an opportunity to significantly reduce the service portfolios and resources spent on some service points, while avoiding highly unpopular closures. Interestingly, some WSCs outlined that every municipality should have physical service provision.

Health centers (tier 1) are the largest centralized, often recently built or renovated, locations that offer comprehensive services (both health and social care) and sometimes also out-of-office hours acute services. Health stations (tier 2) are in moderate to small sized population centers and offer a more restricted but in international comparison still relatively comprehensive portfolio of services. Lastly, service points (tier 3) often offer services greatly limited in scope, for instance only providing nurse appointments once a week. Service points are in the smallest population centers in rural areas and often share a location with a school or other public service (see subheading 3.2.3).

#### 3.1.2. Travel times and distances

Most documents explicitly included travel time and distance information, including the percentage of population reaching services, for instance within 30 minutes by car, before and after the proposed changes. These metrics appear as key indicators for monitoring the accessibility of services, but some more urban WSCs also included data for public transport options.

Some documents explicitly mentioned that the closures and service portfolio reductions should focus on areas with better existing networks. Regardless, transformations of existing health centers to service points occurred mostly in the more rural areas with ageing and decreasing populations.

Many of the reported changes in the proportion of population reaching services were minor, generally ranging from one to four percentage points (for instance a change from 99% to 96%). While these changes appear minor, the number of residents with over 30 minutes of travel by car could increase by thousands per WSC. No documents presented absolute numbers of residents affected.

#### 3.1.3. Accommodating for external factors

The third category encompassed the external factors that the service network planning is aiming to accommodate for, the overarching topic being population characteristics. Workforce and funding are examined under heading 3.2 as justification for changes.

Infrastructure and city developments, ranging from public transport projects to demolition of service centers, were referenced. For instance, many documents included background information about relevant local infrastructure changes and attempted to forecast whether they would result in increased or reduced service needs. Local population mobility patterns and directions were also considered.

Overall, the forecasted service needs appear central to WSCs’ service network planning. Some mentioned that ageing and urbanization trends are already resulting in a downward trajectory of service needs. Furthermore, some WSCs stressed the lingual (including legislative requirements) and cultural needs of the population.

### 3.2 Justifications for changes

#### 3.2.1 Workforce availability

Most WSCs emphasized that the workforce is not sufficient for maintaining service networks inherited from the municipalities, with nearly all explicitly mentioning difficulties in recruiting and retaining nurses and physicians, but also other professionals. Regional analyses indicated that urban areas face fewer recruitment challenges than remote areas, with large discrepancies in workforce availability within the WSCs. Retirements were also highly timely, as large proportions of health workforce are retiring in the coming years.

Small size of service points was seen as a threat to service provision. Vulnerability of smaller units was emphasized, with difficulties in finding temporary or substitutive workforce. In case of absences, turnover, or holiday seasons, these smaller units would be susceptible to temporary closures or cancellation of appointments. Furthermore, recruitment was perceived more difficult in smaller centers, often located in rural areas.

The use of different team models and multidisciplinary care practices require a varied set of professionals, further emphasizing the need for sustained and diverse workforce resources. Lastly, many documents explicitly stated that providing safe working conditions is difficult in smaller centers, where professionals often work alone.

#### 3.2.2 Maintaining high standards of care

High standards of care, referring to accessibility, availability, quality, and efficiency of services, were highlighted as a reason for service network changes. Many WSCs suggested that maintaining high quality and accessible PHC services requires larger sized service points. Centralization was viewed to promote adequate care supply, encompassing both the number and a varied set of professionals. The documents implied that larger centers could better maintain care availability by avoiding appointment cancellations.

Additionally, the efficiency of resource utilization was perceived higher in larger centers. The direct causal pathways were vague, but easier workforce management and interprofessional collaboration were mentioned. Larger centers employ more professionals, providing better opportunities for consultation, collaboration, and specialization.

Quality of care was also referenced, with more experienced staff and better facilities of larger centers leading to higher readiness for complex care conditions. Continuity of care was sometimes better in smaller units, while others argued that centralization promoted continuity. The situation appears differentiated, with health centers enjoying low staff turnover gaining the benefits of continuity, while larger centers might enable the use of ‘personal team’ models.

Lastly, the provision of multidisciplinary care (i.e. integrated services) was mentioned in most documents. As larger centers employ more staff, clients with complex needs can be better assisted. More specialized services are also enabled by higher availability of diagnostics and medical technologies. Some mentioned that clients of smaller centers are often redirected to centralized services, diminishing the usefulness of smaller units.

#### 3.2.3 Premises management and infrastructure

The third central category was the management and optimal utilization of premises and properties. Some WSCs conducted separate premises programs, which were interlinked with service network analyses. A common indicator used was physical surface area of premises per resident, with many explicitly planning reductions. The WSCs emphasized that the service networks were inherited from the municipalities (or a previous joint authority) and that the network of premises needed to be tailored for the current needs.

Most WSCs cited poor condition of premises as one major driver of closures. Many premises were reported as being in ‘poor’ or ‘passable’ condition, requiring complete renovations. Some were also unsuitable for modern working models (e.g. multidisciplinary care). The significant investment needs were often contrasted to forecasted service needs, with many locations deemed unreasonable to invest in.

Some documents mentioned that limited space in the current health centers made centralization efforts difficult. Additionally, digital and mobile care services (see heading 3.3) also require premises. The situation within and between WSCs appears differentiated, with variation in the condition and availability of facilities.

Last, most PHC premises were rented from the municipalities, with national legislation (rent price controls, valuation guidelines) outlining the transition period. However, unclarity following the transition period was mentioned, hampering premise planning efforts beyond year 2026.

#### 3.2.4 The budget

Lastly, we focused on the financial aspects. Interestingly, the widespread budget deficits and needed adjustments were not particularly emphasized as justifications. Some WSCs explicitly mentioned that service network reforms are needed to cover deficits. Others only included a brief mention of the service structure reflecting the received funding, and often in the same sentences other factors (e.g. service workforce challenges) were simultaneously referenced.

Some documents offered detailed numerical data on the planned cost reductions and which domains they target. However, some indicated an upward trajectory in PHC budgets regardless of the service network changes. Many emphasized the overall aim of strengthening PHC services, while conducting significant service network changes and in some cases clear reductions in service provision.

### 3.3. Future of PHC service provision

#### 3.3.1. Digital care

Digital care services were mentioned frequently and linked to extensive growth potential. All WSCs have or are implementing digital clinics, which offer low-threshold consultations and care needs assessment. Digital care has garnered high expectations, with some explicitly outlining an aim, for instance 30–50 percentage of ambulatory outpatient visits being conducted remotely by 2030. Digital services were mentioned as a prerequisite for service network optimization: to close service points, a proportion of current physical consultations must move to digital channels.

Digital care services were framed as an opportunity to increase accessibility and availability of services. As these services are not location dependent, they were viewed to respond to both workforce challenges and the care needs of rural areas. Digital services were also seen to answer the needs of frequent attenders and persons with chronic diseases. Other documents emphasized the importance of ‘multichanneled care’ (i.e. the mix of physical, digital, and mobile care) for regular consultations, while leaving physical services for persons with complex service needs.

#### 3.3.2 Mobile care services

Mobile care services were another central category for the current service network reforms but also for future PHC service provision. These services refer to personnel occasionally mobilizing to rural locations to offer healthcare services. Furthermore, home-based hospital care or vehicles with diagnostic and screening facilities were included.

In WSCs’ views, mobile services enable service provision for remote areas without the requirement of physical premises. Mobile services enable the closure of problematic physical locations while continuing to provide limited services. The need to share premises with a public WSC or municipal service (e.g. school) was recognized. Many WSCs preferred an outreach service model, deploying personnel of centralized units to remote locations, compared to maintaining a small service unit. More flexible workforce management was another implied benefit of mobile services.

Other mobile services, for example vehicles providing diagnostics, screening services, or dental care, were seen to enable the provision of certain specialized care to remote areas, increasing both the accessibility and availability of these services.

#### 3.3.3 Lack of future planning

Lastly, considering the importance of persistent service network planning, we identified a lack of analyses spanning longer than few years. The timeframe of most documents was 2023–2025, with only a few concerning later years. Most WSCs’ documents did not address years beyond 2030, and those that did, were vague.

This might be explained by the hectic reform implementation, with the WSCs simultaneously balancing budgets and harmonizing operations, while not being supported by a long-term vision for the overall healthcare system. Many documents explicitly mentioned that the current changes concerned unifying and integrating services from the previously fragmented structure, including standardization of health centers’ working models and population bases.

Additionally, the four-year council terms (1^st^ from 2023 to 2025) might affect service planning, with most of the documents being drafted during the 1^st^ council term. In general, the current changes were seen as the first version service network reforms, and some documents closed with an explicit mention service network planning is a continuous process and future changes are inevitable.

## 4. Discussion

We examined the WSCs’ principles guiding the service network planning, the justifications provided for service network changes, and views on the future of PHC service networks and service provision. Principles relating to tiered service provision, travel times and distances, as well as accommodating for external, mainly population-related factors, were identified. The WSCs justified the reforms with poor workforce availability, maintaining high standards of care, and premises management. The challenging financial situation was mentioned in some documents, but it was not particularly emphasized. The future of service networks and service provision focused on digital and mobile care services, which were also seen as prerequisites for the physical service network centralization. Last, there was a distinct lack of long-term planning.

In line with the HSPA framework, the WSCs’ documents covered a variety of aspects that guided planning related to PHC service networks. The main functions of resource generation (particularly workforce and premises) as well as access, effectiveness, and quality of service delivery were emphasized, while financing was not addressed comprehensively. This is understandable, as public PHC is mainly provided by the WSCs’ themselves, and such, the role of financing arrangements is not central. Extending the HSPA framework, our results highlight how PHC governance appears to focus also on the contextual determinants, namely population characteristics and forecasted care needs. Aspects of equity related mostly to geographical accessibility and urban-rural divide, while socioeconomic factors were largely absent.

The introduction of a clearly tiered service provision system can offer both the management and the residents of the WSCs a clear expectation for which PHC services are provided where and when, potentially increasing the transparency of service provision. Some WSCs pledged to provide physical services in each municipality – a task made easier with service points that offer reduced portfolio and availability of services. The outlined attempts to avoid focusing changes on areas with existing accessibility challenges were in line with previous quantitative analysis of recent closures [28]. However, areas challenged by poor accessibility and availability of PHC services have been previously identified [37] and their situation following the local network reforms should be monitored. The clearly defined principles outlined in the documents may help the WSCs systematically plan and implement local structural reforms, while minimizing potential negative consequences.

Poor availability of workforce was the most frequently occurring justification for the service network changes. The workforce situation varied between and within the counties, with larger population centers experiencing fewer challenges. This is in line with the previously identified health workforce challenges, both nationally [38] and internationally [4,39], complicating service provision in remote areas. Care availability was perceived worse in smaller units. Hampering the availability and accommodation dimension of accessibility (e.g. opening times) can lead to delayed utilization of services and thus worse health outcomes [7,40], and as such, monitoring the balance between care availability and increased travel times resulting from centralization remains crucial. According to WSCs, larger units can promote continuity of care and integrated multidisciplinary services. While full organizational integration is not a pre-requisite of integrated care, providing comprehensive co-located services can help facilitate collaboration between professionals and link together traditionally separated health and social care [41,42].

The documents paint a worrying picture of the current PHC network, with facilities in poor technical condition. By 2030, the investment needs for PHC facilities are estimated to be nearly 860 million euros nationwide [43]. As a comparison, the total PHC expenditures for 2024 were approximately 4 billion euros. The approaching structural reform might have left the municipalities with little incentive to invest in their premises, an issue compounded by the reform delays. In addition, a lack of long-term vision for the PHC system, including the structure of organization and service models, might have further hampered the planning efforts of public actors. Many of the renovation needs concentrated on rural areas with declining populations and peaking service needs, further disincentivizing investments. These findings highlight the importance of long-term planning of both health systems and service organization, also during reform preparation and implementation, as future PHC services might require different types and locations of facilities.

The financial aspects were not especially emphasized in the WSCs service network analyses. However, budgets, rebalancing needs, and adjustments by service type are largely covered by other WSCs’ documents, such as the various productivity and efficiency programs, which were not examined in this study. Nevertheless, the lack of attention to financial challenges as justifications for the changes underlines the importance of the other, persisting factors, which are also driving the local reforms. As such, it is likely that some proportion of the current PHC service network changes would have been implemented regardless of the financial situation. It appears that the changes are met with a general feeling of austerity and worry among the population [29], while the WSCs view the inherited service networks as outdated and in need of reconfiguration to safeguard the sustainability of future service provision.

Digital and mobile services are a key part of the WSCs’ future PHC service networks and service provision, and as a prerequisite for their physical service network reforms. Previous research has identified various identified barriers [44,45] particularly relevant for the ageing population in rural areas. Consequently, the WSCs must adequately support service use and safeguard opportunities to access traditional service provision when needed, especially as the suitability of digital services as a replacement for physical services remains unclear [46]. Mobile care (i.e. outreach service model) can help provide low-cost services to at-risk and remote populations [47,48] and offer a way to provide limited services without maintaining physical facilities [49]. This also has implications for alleviating health workforce challenges. Lastly, the documents suggest that the timeframe of service network planning is currently short, which could be explained by the large structural reform’s rapid implementation, but also by the four-year terms of the elected county councils. To facilitate reaching the national aim of strengthening PHC services the WSCs should strive for stability and continuity in the service network planning process.

Contrasting our findings with the Nordic core values and principles of general practice [50] reveals distinct differences in focus. The Finnish WSCs appear to mainly concentrate on improving availability and access through centralization and digital services, which remain absent in the Nordic core values. Through centralization, the WSCs attempt to promote continuity of care and multidisciplinary collaboration, which as aims align with the Nordic core values 1 and 7. However, improving continuity of care by closing service points and centralization is an approach not supported by current evidence [51,52]. Several values, particularly person-centered medicine (4), education and research (5), as well as equity and social strain (6) did not emerge in the network planning documents of WSCs, underlining the focus of the service network planning (i.e. availability and accessibility of care) not being fully in line with the overall general aims of PHC.

### 4.1. Practical implications and future research

The categories emerging from the documents suggest that the service network planning processes of the new WSCs are thorough and data-driven, encompassing a diverse set of factors, ranging from population characteristics to infrastructure projects. These findings align with various health planning frameworks [53] and the WHO Operational Framework for PHC [54], which also underline the importance of workforce, infrastructure, and effective models of care. The Finnish service delivery models centered around tiered service provision and digital and mobile care services are presented as real-life examples of maintaining accessibility during service centralization.

In line with previous analyses of regional PHC planning [14] and the HSPA framework [31], our results highlight the complexity and interconnectedness of service network planning. The identified principles of local planning can help decision-makers evaluate how and what evidence is used, identify potential gaps, and ultimately improve their planning processes to promote evidence-informed decision-making. Policy and system researchers can incorporate our findings into subsequent research examining public service networks in other settings also facing centralization, such as hospitals or nursing homes.

Internationally, our results are likely of interest by countries conducting similar structural reforms, such as Denmark [55] and Ireland [56]. New actors in these reforms might need to restructure or re-examine their service networks if not immediately, then in the future, driven by global urbanization trends, workforce challenges, and growing efficiency demands. Additionally, some countries are planning or have implemented reforms for which our analysis is pertinent. Examples include England’s integrated care systems [57] and Austria’s plan to triple the number of PHC centers [58]. Comprehensive planning is imperative to ensure optimized service networks that best promote population health under the given resource constraints [31]. While the use of various priority-setting methods and approaches can potentially assist in effective resource-allocation [59], the complexity and contested nature of local service provision must be accounted for [60,61].

Some topics were identified in the documents that warrant further research. First, while population sentiment and outcomes were outside the scope of this study, the importance of monitoring the impact of the current PHC service network changes on care accessibility and service utilization cannot be overstated. While the WSCs framed the changes as necessary and in some cases beneficial, accessibility must be monitored, especially among vulnerable groups and remote populations. Closure of physical service points can undermine and erode trust in the health system [29]. Providing opportunities for civic participation can help maintain and restore population confidence in the services [62].

This research highlighted the potential of examining public policy documents. The current service network changes also concerned social care and specialized services, and similar analysis of principles and justifications could be conducted for other care settings and contexts. These concurrent changes and their effects might consequently accumulate on some population groups or geographical areas, underlining the need for thorough impact and risk assessment, evaluation, and research.

### 4.2. Limitations

This research draws from the authors’ backgrounds in social and health sciences, namely public health, health management sciences, and health policy. The authors are employed at a national research institute, which has a function in evaluating and information steering of WSCs, but this research is not associated with these functions. As qualitative research is reflexive in nature [63], it is possible that other researchers with different (professional) backgrounds could have arrived at different conclusions. To strengthen credibility and trustworthiness, we formulated clear research questions, utilized predefined inclusion criteria, iteratively and collaboratively refined our results, and presented illustrative excerpts from the documents. Additionally, only publicly available documents were included, leaving out information from private documents (e.g. internal memos). Next, the data used consisted of WSCs’ own reports, and as such, the planning and decision-making could be framed in a more positive light. However, the documents are drafted by public servants who have liability while in office, supporting their credibility. Additional research providing data and methodological triangulation is, however, needed to confirm our findings.

The policy documents examined concern the first three years of a large-scale structural reform. As such, our findings are likely shaped by reform implementation, the challenging operating environment, and financial difficulties. This limits the transferability of our results to service network planning occurring in non-reform contexts. Furthermore, the public PHC services provided in health centers, a relatively uncommon organization model in Europe, challenges transferability to systems relying on private GP clinics. Regardless, this study can inform planning and implementation of comparable structural reforms, also beyond the present context.

## 5. Conclusions

Following a large-scale administrative reform, the PHC service network planning of the new Finnish WSCs is guided by multiple factors, comprising tiered service provision models with adequate travel times, while accounting for population characteristics. Justifications for closures focused on workforce availability, condition of premises, and ensuring high standards of care. Future care provision relies increasingly on digital and mobile care services, aiming to maintain adequate access and availability in remote areas. These novel results highlight the complexity of service network planning during reform implementation and in an extraordinarily challenging operating environment.

## Data Availability

The datasets used and/or analyzed during the current study are available from the corresponding author on reasonable request.

## References

[1] World Health Organization. A vision for primary health care in the 21st century: towards universal health coverage and the Sustainable Development Goals. Geneva: World Health Organization and the United Nations Children’s Fund (UNICEF); 2018.

[2] Bloom DE, Chatterji S, Kowal P, et al. Macroeconomic implications of population ageing and selected policy responses. Lancet. 2015;385(9968):649–657. doi: 10.1016/S0140-6736(14)61464-1. 25468167 PMC4469267

[3] Kadakia KT, Galea S. Urbanization and the future of population health. Milbank Q. 2023;101(S1):153–175. doi: 10.1111/1468-0009.12624. 37096620 PMC10126956

[4] Boniol M, Kunjumen T, Nair TS, et al. The global health workforce stock and distribution in 2020 and 2030: a threat to equity and ‘universal’ health coverage? BMJ Glob Health. 2022;7(6):e009316. doi: 10.1136/bmjgh-2022-009316. PMC9237893 35760437

[5] Hacker K. The burden of chronic disease. Mayo Clin Proc Innov Qual Outcomes. 2024;8(1):112–119. doi: 10.1016/j.mayocpiqo.2023.08.005. 38304166 PMC10830426

[6] World Health Organization. World mental health report: transforming mental health for all [Internet]. Geneva: World Health Organization; 2022. Available from: https://www.who.int/publications/i/item/9789240049338

[7] Levesque JF, Harris MF, Russell G. Patient-centred access to health care: conceptualising access at the interface of health systems and populations. Int J Equity Health. 2013;12(1):18. doi: 10.1186/1475-9276-12-18. 23496984 PMC3610159

[8] Penchansky R, Thomas JW. The concept of access: definition and relationship to consumer satisfaction. Med Care. 1981;19(2):127–140. doi: 10.1097/00005650-198102000-00001. 7206846

[9] Kringos DS, Boerma WG, Hutchinson A, et al. Building primary care in a changing Europe. The United Kingdom: World Health Organization. Regional Office for Europe; 2015.

[10] OECD. Realising the Potential of Primary Health Care [Internet]. OECD Health Policy Studies, OECD Publishing, Paris; 2020. [cited 2023 May 11]. (OECD Health Policy Studies, OECD Publishing, Paris). Available from: https://www.oecd-ilibrary.org/social-issues-migration-health/realising-the-potential-of-primary-health-care_a92adee4-en doi: 10.1787/a92adee4-en.

[11] Holte JH, Kjaer T, Abelsen B, et al. The impact of pecuniary and non-pecuniary incentives for attracting young doctors to rural general practice. Soc Sci Med. 2015;128:1–9. doi: 10.1016/j.socscimed.2014.12.022. 25569609

[12] Ozegowski S. Effective policy mechanisms for an equitable geographical distribution of general practitioners: a qualitative comparative analysis of the accessibility of primary care in Europe. J Health Serv Res Policy. 2013;18(3):151–159. doi: 10.1177/1355819613482885. 23864419

[13] Denmark BDO. Kortlægning af udvikling i praksisformer i almen praksis [Mapping the developments of practices in general practice] [Internet]; 2023. Available from: https://www.ism.dk/Media/638344438524356297/01Kortlægning%20af%20udvikling%20i%20praksisformer%20i%20almen%20praksis%20-%20august%202023.pdf.

[14] Windle A, Javanparast S, Freeman T, et al. Factors that influence evidence-informed meso-level regional primary health care planning: a qualitative examination and conceptual framework. Health Res Policy Syst. 2023;21(1):99. doi: 10.1186/s12961-023-01049-8. 37749644 PMC10521552

[15] Windle A, Javanparast S, Freeman T, et al. Use of evidence to inform regional primary health care planning in Australia. Health Res Policy Syst. 2025;23(1):31. doi: 10.1186/s12961-025-01308-w. 40069702 PMC11900053

[16] Ahmadi-Javid A, Seyedi P, Syam SS. A survey of healthcare facility location. Computers & Operations Research. 2017;79:223–263. doi: 10.1016/j.cor.2016.05.018.

[17] Fränti P, Sieranoja S, Laatikainen T. Designing a clustering algorithm for optimizing health station locations. Int J Health Geogr. 2025;24(1):4. doi: 10.1186/s12942-025-00390-1. 40121476 PMC11929219

[18] Tynkkynen LK, Keskimäki I, Karanikolos M. Finland: Health System Summary, 2024 [Internet]. 2024;Copenhagen: WHO Regional Office for Europe on behalf of the European Observatory on Health Systems and Policies. Available from: https://iris.who.int/bitstream/handle/10665/379912/9789289014410-eng.pdf?sequence=1.

[19] Jokelin E, Piirainen L, Nissen M, et al. Healthcare cost trajectories with a multidisciplinary team model: a three-year follow-up from Finnish primary and secondary care. Scand J Prim Health Care. 2026;44(1):2650552. doi: 10.1080/02813432.2026.2650552. 41965256 PMC13072694

[20] Eskola P, Tuompo W, Riekki M, et al. Hoidon jatkuvuusmalli: Omalääkäri 2.0 -selvityksen loppuraportti [Sosiaali- ja terveysministeriön raportteja ja muistioita] [Internet]. 2022. Sosiaali- ja terveysministeriön raportteja ja muistioita no.: 17. Available from: http://urn.fi/URN:ISBN:978-952-00-9884-1.

[21] Holster T, Nguyen L, Häkkinen U. The role of occupational health care in ambulatory health care in Finland. Nordic J Health Eco. 2022;10(1):61–81. doi: 10.5617/njhe.8561.

[22] Wahlbeck K, Manderbacka K, Vuorenkoski L, et al. Quality and equality of access to healthcare services: HealthQUEST country report for Finland; 2008.

[23] Hernández-Quevedo C, Maresso A, Merkur S, et al. 20 years of health system reforms in Europe: what’s new? Eurohealth (Lond). 2018;24(2):23–28.

[24] Paatela S, Huhtakangas M, Tynkkynen LK. Governance of a health and social service system after two years of a large-scale reform: a qualitative study in Finland. J Health Organ Manag. 2026;40(2):254–270. doi: 10.1108/JHOM-01-2025-0055. 40900619

[25] Lumme S, Satokangas M, Holster T. https://www2.eduskunta.fi/FI/naineduskuntatoimii/julkaisut/Documents/TrV_1_2025.pdf., et al. Perustason avosairaanhoidon rakenteet, resurssit ja rahoitus [Structures, resources and financing of outpatient primary care]. Eduskunnan tarkastusvaliokunnan julkaisu [Internet]. 2025;(1). Available from:

[26] Act on Wellbeing Services Counties [Internet]. 611/2021; 2021 Jun 30. Available from: https://www.finlex.fi/fi/lainsaadanto/2021/611.

[27] OECD. Country Health Profile 2025: Finland [Internet]. OECD Publishing, Paris/European Observatory on Health Systems and Policies, Brussels; 2025. (State of Health in the EU). Available from: https://eurohealthobservatory.who.int/publications/m/finland-country-health-profile-2025.

[28] Väisänen V, Tynkkynen LK, Lavaste K, et al. Primary health center unit closures following the Finnish administrative reform: a multilevel analysis of determinants. medRxiv. 2025. doi: 10.1101/2025.11.17.25340376.

[29] Kihlström L, Keskimäki I, Paananen H, et al. How is a large-scale reform perceived by citizens in Finnish primary health care? Soc Sci Med. 2025;387:118688. doi: 10.1016/j.socscimed.2025.118688. 41110247

[30] Kihlström L, Viita-Aho M, Keskimäki I, et al. How health workers experience health system reform: A qualitative study from Finland. Health Policy (New York). 2025;163(1):105499. doi: 10.1016/j.healthpol.2025.105499. 41273901

[31] Rajan D, Papanicolas I, Karanikolos M. others. Health System Performance Assessment: a Renewed Global Framework for Policy-Making. Geneva, WHO Regional office for Europe (On Behalf of the European Observatory on Health Systems and Policies). 2023.

[32] World Health Organization. Everybody’s business: strengthening health systems to improve health outcomes: WHO’s framework for action. Geneva: World Health Organization; 2007.

[33] Greer S, Wismar M, Figueras J. Strengthening health system governance: better policies, stronger performance. England, Open University Press, European Observatory on Health Systems and Policies series. 2015.

[34] Bowen GA. Document analysis as a qualitative research method. Qualitative Research Journal. 2009;9(2):27–40. doi: 10.3316/QRJ0902027.

[35] Elo S, Kyngäs H. The qualitative content analysis process. J Adv Nurs. 2008;62(1):107–115. doi: 10.1111/j.1365-2648.2007.04569.x. 18352969

[36] Dalglish SL, Khalid H, McMahon SA. Document analysis in health policy research: the READ approach. Health Policy Plan. 2021;35(10):1424–1431. doi: 10.1093/heapol/czaa064. 33175972 PMC7886435

[37] Väisänen V, Satokangas M, Huhtakangas M, et al. Medical deserts in Finland: measuring the accessibility and availability of primary health care services. BMC Health Serv Res. 2025;25(1):281. doi: 10.1186/s12913-025-12409-1. 39972338 PMC11841193

[38] Kirkonpelto TM, Mäntyranta T editors. Tiekartta 2022–2027: sosiaali- ja terveysalan henkilöstön riittävyyden ja saatavuuden turvaaminen [Roadmap for 2022–2027Ensuring the sufficiency and availability of healthcare and social welfare personnel]. Helsinki. 2023; Sosiaali- ja terveysministeriön julkaisuja 2023;8.

[39] World Health Organization. Global strategy on human resources for health: workforce 2030 [Internet]. Geneva: World Health Organization; 2016. [cited 2023 Jun 13]. 64 p. Available from: https://apps.who.int/iris/handle/10665/250368

[40] Rosano A, Loha CA, Falvo R, et al. The relationship between avoidable hospitalization and accessibility to primary care: a systematic review. Eur J Public Health. 2013;23(3):356–360. doi: 10.1093/eurpub/cks053. 22645236

[41] Lalani M, Marshall M. Co‐location, an enabler for service integration? Lessons from an evaluation of integrated community care teams in East London. Health Soc Care Community. 2022;30(2):e388–e396. doi: 10.1111/hsc.13211. 33152144 PMC9290730

[42] Valentijn PP, Schepman SM, Opheij W, et al. Understanding integrated care: a comprehensive conceptual framework based on the integrative functions of primary care. Int J Integr Care. 2013;13(1):e010. doi: 10.5334/ijic.886. 23687482 PMC3653278

[43] Maakuntien tilakeskus. Selvitys hyvinvointialueiden tilainvestoinneista [Internet]. Helsinki. 2025; Available from: https://www.maakuntientilakeskus.fi/wp-content/uploads/2025/06/11062025_Maakuntien-tilakeskus_Selvitys-hyvinvointialueiden-tilainvestoinneista.pdf

[44] Kruse C, Fohn J, Wilson N, et al. Utilization barriers and medical outcomes commensurate with the use of Telehealth among older adults: systematic review. JMIR Med Inform. 2020;8(8):e20359. doi: 10.2196/20359. 32784177 PMC7450384

[45] Heponiemi T, Kaihlanen A-M, Virtanen L, et al. The mediating role of digital competence in the associations between the factors affecting healthcare utilization and access to care. Int J Public Health. 2023;68:1606184. doi: 10.3389/ijph.2023.1606184. 38250321 PMC10796446

[46] Lewinski AA, Walsh C, Rushton S, et al. Telehealth for the longitudinal management of chronic conditions: systematic review. J Med Internet Res. 2022;24(8):e37100. doi: 10.2196/37100. 36018711 PMC9463619

[47] Hill CF, Powers BW, Jain SH, et al. Mobile health clinics in the era of reform. Am J Manag Care. 2014;20(3):261–264. 24884754

[48] Yu SWY, Hill C, Ricks ML, et al. The scope and impact of mobile health clinics in the United States: a literature review. Int J Equity Health. 2017;16(1):178. doi: 10.1186/s12939-017-0671-2. 28982362 PMC5629787

[49] Roodenbeke E d, Lucas S, Rouzaut A, et al. Outreach Services as a Strategy to Increase Access to Health Workers in Remote and Rural Areas: increasing Access to Health Workers in Rural and Remote Areas [Internet]. Geneva: World Health Organization; 2011. [cited 2026 Jun 1]. (WHO Guidelines Approved by the Guidelines Review Committee). Available from: http://www.ncbi.nlm.nih.gov/books/NBK310729/ PubMed PMID: 26269879. 26269879

[50] Nordic Federation of General Practice (NFGP). Core values and principles of Nordic General Practice/Family Medicine. Scand J Prim Health Care. 2020;38(4):367–368. doi: 10.1080/02813432.2020.1842674. 33284030 PMC7782180

[51] Simonsen M, Skipper L, Skipper N, et al. Discontinuity in care: practice closures among primary care providers and patient health care utilization. J Health Econ. 2021;80:102551. doi: 10.1016/j.jhealeco.2021.102551. 34785433

[52] Forbes LJ, Forbes H, Sutton M, et al. Changes in patient experience associated with growth and collaboration in general practice: observational study using data from the UK GP Patient Survey. Br J Gen Pract. 2020;70(701):e906–15–e915. doi: 10.3399/bjgp20X713429. 33139333 PMC7643819

[53] Gudes O, Kendall E, Yigitcanlar T, et al. Rethinking health planning: a framework for organising information to underpin collaborative health planning. Health Inf Manag. 2010;39(2):18–29. doi: 10.1177/183335831003900204. 20577020

[54] World Health Organization. Operational framework for primary health care: transforming vision into action; 2020.

[55] Birk HO, Vrangbæk K, Rudkjøbing A, et al. Denmark: health system review. Health Syst Transit. 2024;26(1):1–186. 38841877

[56] Burke S, Barry S, Siersbaek R, et al. Sláintecare – A ten-year plan to achieve universal healthcare in Ireland. Health Policy. 2018;122(12):1278–1282. doi: 10.1016/j.healthpol.2018.05.006. 29843901

[57] Anderson M, Pitchforth E, Edwards N, et al. United Kingdom: health system review; 2022. 35579557

[58] Bachner F, Bobek J, Habimana K, et al. Austria: Health System Summary, 2024 [Internet]. 2024;Copenhagen: WHO Regional Office for Europe on behalf of the European Observatory on Health Systems and Policies. Available from: https://iris.who.int/bitstream/handle/10665/379901/9789289014335-eng.pdf.

[59] Mitton C, Donaldson C. Health care priority setting: principles, practice and challenges. Cost Eff Resour Alloc. 2004;2(1):3. PubMed Central PMCID: PMC411060. doi: 10.1186/1478-7547-2-3. 15104792 PMC411060

[60] McDonald J, Ollerenshaw A. Priority setting in primary health care: a framework for local catchments. Rural Remote Health. 2011;11(2):1714. 21657809

[61] Daniels N. Accountability for reasonableness. BMJ. 2000;321(7272):1300–1301. doi: 10.1136/bmj.321.7272.1300. 11090498 PMC1119050

[62] Djellouli N, Jones L, Barratt H, et al. Involving the public in decision-making about large-scale changes to health services: a scoping review. Health Policy. 2019;123(7):635–645. doi: 10.1016/j.healthpol.2019.05.006. 31147108

[63] Olmos-Vega FM, Stalmeijer RE, Varpio L, et al. A practical guide to reflexivity in qualitative research: AMEE Guide No. 149. Med Teach. 2022;45(3):1–11. doi: 10.1080/0142159X.2022.2057287. 35389310

